# Mesoporous hydrochar from *Acacia falcata* leaves by hydrothermal process for hexavalent chromium adsorption

**DOI:** 10.1038/s41598-025-96439-z

**Published:** 2025-04-12

**Authors:** Rajesh Juturu, Ramesh Vinayagam, Gokulakrishnan Murugesan, Raja Selvaraj

**Affiliations:** 1 Department of Biotechnology, Manipal Institute of Technology, Manipal Academy of Higher Education, Manipal, Karnataka 576104 India; 2 Department of Chemical Engineering, Manipal Institute of Technology, Manipal Academy of Higher Education, Manipal, Karnataka 576104 India; 3 Department of Biotechnology, M.S. Ramaiah Institute of Technology, Bengaluru, Karnataka 560054 India

**Keywords:** Adsorption, Hexavalent chromium, Hydrochar, Regeneration studies, Spiking studies, Environmental sciences, Nanoscale materials, Chemical engineering, Chemical engineering, Environmental chemistry, Green chemistry, Materials chemistry

## Abstract

This study evaluates mesoporous-hydrochar derived from *Acacia falcata* leaves *via* a single-step hydrothermal treatment for Cr(VI) adsorption. Material characterization indicated that the adsorbent has a rough and porous structure. FTIR analysis confirmed Cr(VI) adsorption through functional group interactions, evidenced by peak intensity changes and the emergence of a Cr–O bond vibration at 669 cm^-1^. Two new peaks were observed in XPS spectra, corresponding to Cr 2p at 577.04 eV (Cr 2p3/2) and 586.67 eV (Cr 2p1/2) after adsorption, further substantiating the adsorption and Cr(VI) reduction. Batch experiments showed an improved adsorption capacity of 30.47 mg/g. Kinetic investigation adhered to the pseudo-second-order model, whereas the equilibrium dataset satisfied the Freundlich model, indicating a heterogeneous adsorption mechanism involving physisorption and chemisorption. The thermodynamic evaluation confirmed spontaneous and endothermic adsorption. Regeneration studies showed reduced Cr(VI) removal performance after four cycles, attributed to pore blockage and loss of functional groups while maintaining effective reuse potential. Spiked studies in various water matrices showed a slight decrease in Cr(VI) removal efficiency, yet it maintained over 95% efficiency, demonstrating its potential for real-world water treatment applications.

## Introduction

The rapid growth of industrialization, coupled with an unprecedented surge in global population, has significantly compromised the quality of water resources worldwide. As industries expand and urbanization accelerates, the volume and complexity of wastewater generated have increased substantially, containing a wide array of harmful contaminants that endanger aquatic ecosystems and human health^1^. Heavy metal contaminants like cadmium, chromium, mercury, arsenic, and lead, are particularly problematic as they do not degrade naturally and tend to accumulate over time. Amongst these contaminants, hexavalent chromium, Cr(VI), is notably more notorious than its trivalent counterpart. Long-term exposure to trace levels of Cr(VI) is related to various health problems, including allergic reactions, hereditary genetic defects, and cancer^2^. Its ability to enter biological systems through ingestion, inhalation, or skin contact makes it a persistent health hazard. Chromium is widely utilized across various manufacturing processes, like electroplating, tanning, metallurgy, and printing^3^. These industries generate significant amounts of wastewater, which frequently carries elevated levels of Cr(VI). For instance, the leather tanning industry alone discharges between 30 and 35 L of water containing Cr(VI) for every kilogram of leather produced, contributing significantly to environmental pollution^4^.

Various methods have been employed for treating Cr(VI) contamination in waterbodies, each offering distinct advantages and limitations based on efficiency, cost, and environmental impact^5^. Chemical precipitation converts Cr(VI) into an insoluble form for removal via sedimentation or filtration but generates sludge requiring further treatment. Ion exchange employs resins to capture Cr(VI) selectively, though competing ions can reduce efficiency. Reverse osmosis and ultrafiltration efficiently remove Cr(VI) using semi-permeable membranes but demands frequent maintenance due to membrane fouling. Among the available techniques, adsorption stands out as a competent approach due to its simplicity, affordability, improved removal performance, and the availability of various adsorbents, including activated carbon, magnetic biochar, hydrochar, nanomaterials, and modified nanomaterials^6^. Selecting a suitable remediation technique depends on several factors, including Cr(VI) concentration, water matrix composition, operational costs, and environmental sustainability. Among the adsorbents, hydrochar serves as an effective option to remediate Cr(VI) due to its affordability, sustainability, and superior adsorption efficiency. It is a carbon-dense substance generated via the thermochemical transformation of organic matter in the presence of water at moderate temperatures^7^.

Hydrochar can be synthesized through microwave-assisted synthesis, muffle furnace pyrolysis, and hydrothermal carbonization (HTC). While microwave synthesis is rapid and energy-efficient, muffle furnace pyrolysis is slower and more energy-intensive^8^. Whereas, hydrochar is synthesized through HTC, a thermochemical method that transforms biomass into a carbon-rich substance by utilizing water as the medium at a modest temperature (180–250 °C) and self-generated pressure (2–10 MPa)^9^. The process begins with hydrolysis, where biomass polymers such as cellulose, hemicellulose, and lignin break down into smaller organic molecules, including sugars, acids, and furans^10^. These intermediates then undergo dehydration, resulting in the elimination of hydroxyl functional moieties and the formation of carbonyl and furan derivatives^11^. Next, decarboxylation and deamination remove carboxyl and amine groups as CO_2_ and NH_3_, respectively, resulting in highly reactive oxygenated intermediates^12^. These intermediates further undergo polymerization and aromatization, forming a carbonaceous network with enriched carbon content^13^. The final hydrochar retains oxygen-holding moieties, containing carboxyl, hydroxyl, and carbonyl, which improves its adsorption capacity for Cr(VI) removal. Additionally, HTC offers improved control over physicochemical characteristics, such as surface area, porosity, and hydrophilicity, by modifying reaction conditions. Furthermore, its characteristics can be further optimized through acid/base treatments, metal impregnation, or activation to enhance porosity and surface functionality. Moreover, the hydrochar produced via HTC offers advantages over activated carbon, including reduced energy requirements due to its lower operating temperature and the ability to process wet biomass directly, eliminating the need for drying^14^. HTC also requires fewer chemicals and has a higher yield compared to activated carbon, making it more resource-efficient.

Despite the growing interest in HTC for synthesizing carbonaceous absorbents, limited studies have explored the HTC of leaf biomass for Cr(VI) remediation. Previous studies have explored the HTC of other biomass sources for heavy metal adsorption. For instance, Nhambe et al.^15^ prepared an adsorbent from pulp and paper sludge and used it for lead adsorption. Yildirir^16^ synthesized vermicompost hydrochar for methylene blue dye removal. In our earlier study, magnetic hydrochar was synthesized through an HTC process and used to remove Cr(VI), achieving an adsorption efficiency of 36.15 mg/g^17^.

The novelty of this study lies in using hydrochar synthesized from *Acacia falcata* leaves as an efficient and sustainable adsorbent for Cr(VI) removal. This approach is relatively unexplored, offering a cost-effective and eco-friendly alternative to conventional adsorbents. Unlike activated carbon and modified hydrochar, which require expensive chemicals, high temperatures, and complex synthesis steps, hydrochar is produced in a single step with minimal resources, making it a suitable adsorbent for Cr(VI) remediation. This study also examines the regeneration potential of hydrochar to assess its reusability and cost-effectiveness, an aspect that is scarcely studied for Cr(VI) removal. Additionally, the effect of competing ions commonly found in real wastewater on Cr(VI) adsorption is evaluated, addressing another underexplored area.

Therefore, the main objectives of this research are : (1) synthesis of hydrochar from *A. falcata* leaves powder using HTC process, (2) physiochemical characterization using various analytical instruments, (3) effects of various parameters via batch studies, (4) kinetic, isotherm, and thermodynamic models are employed to investigate the interactions between Cr(VI) and HC binding sites.(5) regeneration studies of HC and (6) the effect of competing ions present in various water matrices on Cr(VI) removal using synthesized HC is examined.

## Experimental sections

### Chemicals used

Analytical-grade chemicals were employed directly without the need for further purification. HCl (35.4%), NaOH (97%), and H_2_SO_4_ (96%) were sourced from Loba Chemie, India. Furthermore, acetone (99%), 1,5-diphenylcarbazide (98%), and K_2_Cr_2_O_7_ (99%) were obtained from Merck, Germany.

### Preparation of hydrochar using *A. falcata* leaves

*A. falcata* leaves, collected from the MIT campus in Manipal, India, were meticulously washed with distilled water. After washing, the leaves were evenly spread and heated in an air circulation oven set at 353 K to remove any remaining moisture. After being thoroughly dried, the leaves were finely ground into a uniform powder using a mixer grinder and stored in an airtight vessel to prevent moisture absorption. The *A. falcata* hydrochar was synthesized using a modified version of the method employed by Pan et al. for almond shell hydrochar^18^. Initially, 5 g of *A. falcata* leaf powder was mixed with 30 mL of water in a 250 mL container and agitated for 15 min to form a uniform slurry. The prepared slurry was later transferred into a Teflon-lined hydrothermal reactor and placed in a hot air oven and heated at 473 K, for 600 min. After cooling, the solid product was washed with distilled water and later heated in an air circulation oven at 353 K to eliminate residual moisture. The adsorbent thus synthesized from *A. falcata* leaves, labeled as AFHC, was utilized for Cr(VI) remediation.

### Characterization instruments

Fourier transform infrared spectroscopy (FTIR) was done using equipment from Agilent Technologies, USA, to examine the surface functional groups of AFHC. The Brunauer-Emmett-Teller (BET) method was used to determine the specific surface area (SSA), pore volume, and pore size distribution using a BELSORP-miniX instrument from MicrotracBEL, Japan. The samples were analyzed through N_2_ adsorption and desorption at 77 K. The elemental nature and surface morphological features were examined using an Oxford Instrument system, UK, and a Neon 40 microscope from Carl Zeiss, Germany. The crystallinity of AFHC was determined through XRD analysis using a D8 Advance instrument from Bruker, Germany. X-ray photoelectron spectroscopy (XPS) was done using the K-Alpha 149 instrument from Thermo Fisher Scientific, UK, facilitating the examination of the elemental nature.

### Batch experimentation tests

Batch adsorption was performed to assess the Cr(VI) adsorption from water samples. A Cr(VI) stock was made by dissolving K_2_Cr_2_O_7_ in distilled water and diluted to achieve the required composition. The solution pH (2–12) was adjusted using HCl or NaOH. The AFHC dosage varied between 0.1 and 0.8 g/L to examine its influence on Cr(VI) removal. Initial Cr(VI) concentration was varied between 5 and 25 mg/L. The rate of adsorption was experimented with by varying the adsorption time from 5 to 180 min. Samples were withdrawn at predetermined intervals for analysis. The impact of temperature was investigated between 293 and 323 K to evaluate thermodynamic constants. Following the adsorption process, residual Cr(VI) was analyzed using UV-visible spectrophotometry at 540 nm after reacting with DPC. The adsorption capacity (q_e_) and removal efficiency (%) were calculated by Eqs. (1) and (2).

1 $$\:{q}_{e}=\frac{({C}_{i}-{C}_{f})}{{m}_{AFHC}}\times\:V$$

2 $$\:R\left(\%\right)=\frac{\left({C}_{i}-{C}_{f}\right)}{{C}_{i}}\times\:100$$

wherein, C_i_ and C_f_ denote the respective starting, and final Cr(VI) concentration remaining at a specific time, measured in mg/L. V signifies the solution volume in liters (L), and m_AFHC_ indicates the AFHC quantity in g.

Batch adsorption data were evaluated through isotherm, kinetic, and thermodynamic model equations to elucidate the interaction between Cr(VI) and AFHC. Kinetic models that include Pseudo First-Order (PFO), Pseudo Second-Order (PSO), and Intra-particle Diffusion (IPD), were employed to evaluate the rate-limiting step^19^. Isotherm models, like Langmuir, Freundlich, and Temkin, described the equilibrium behavior and adsorption capacity^20^. Thermodynamic analysis assessed the process’s spontaneity, enthalpy, and entropy^21^.

### Regeneration studies

The reusability of AFHC for Cr(VI) removal was assessed through multiple adsorption-desorption cycles. A 0.1 N NaOH solution was chosen as the regeneration eluent because maximum chromium removal occurred under acidic conditions (pH 2 to 4), where strong electrostatic interactions prevailed between the positively charged AFHC surface and negatively charged Cr(VI) species. In contrast, basic conditions proved effective for regeneration, as hydroxyl ions displaced the adsorbed Cr(VI) ions, preserving AFHC’s adsorption capacity across several cycles. Specifically, a 0.1 N NaOH solution was selected due to its pH of approximately 13.

In our previous work, magnetic biochar was employed for Cr(VI) removal and regenerated using 0.1 N and 0.5 N NaOH solutions. The 0.1 N solution outperformed the higher concentration, possibly because elevated ionic strength led to the loss of surface functional groups^17^. Numerous studies have similarly identified alkaline conditions as effective for adsorbent regeneration, often utilizing a 0.1 M NaOH solution^22,23^. Consequently, in this study, a 0.1 N NaOH solution was adopted as the regenerating eluent. So, regeneration studies were carried out in conical flask with 100 mL Cr(VI) solution, with the pH adjusted to 2, AFHC of 1.2 g/L at 303 K under continuous agitation at 150 rpm in an orbital shaker for 180 min. Post-adsorption the spent AFHC was regenerated by treating it with 0.1 N NaOH (50 mL), 120 min, followed by rinsing with deionized water and drying before reuse in subsequent cycles. The reusability of AFHC for Cr(VI) removal was assessed through multiple adsorption-desorption cycles.

### Application of AFHC in Cr(VI) removal from surface and groundwater

Batch sorption experiments were performed by spiking Cr (VI) into different water matrices to assess the impact of interfering ions on adsorption efficiency. Water samples from groundwater, Manipal Lake, tap water, and Swarna River, were collected to evaluate the influence of inherent water constituents on Cr(VI) removal. For each batch experiment, 100 mL of 10 mg/L Cr(VI) was used with 1.2 g/L of AFHC at pH 2. Adsorption was carried out at 303 K under constant agitation at 150 rpm in an orbital shaker for 180 min.

## Results and discussion

### Characterization

#### FTIR and XRD analyses

**Fig. 1:**
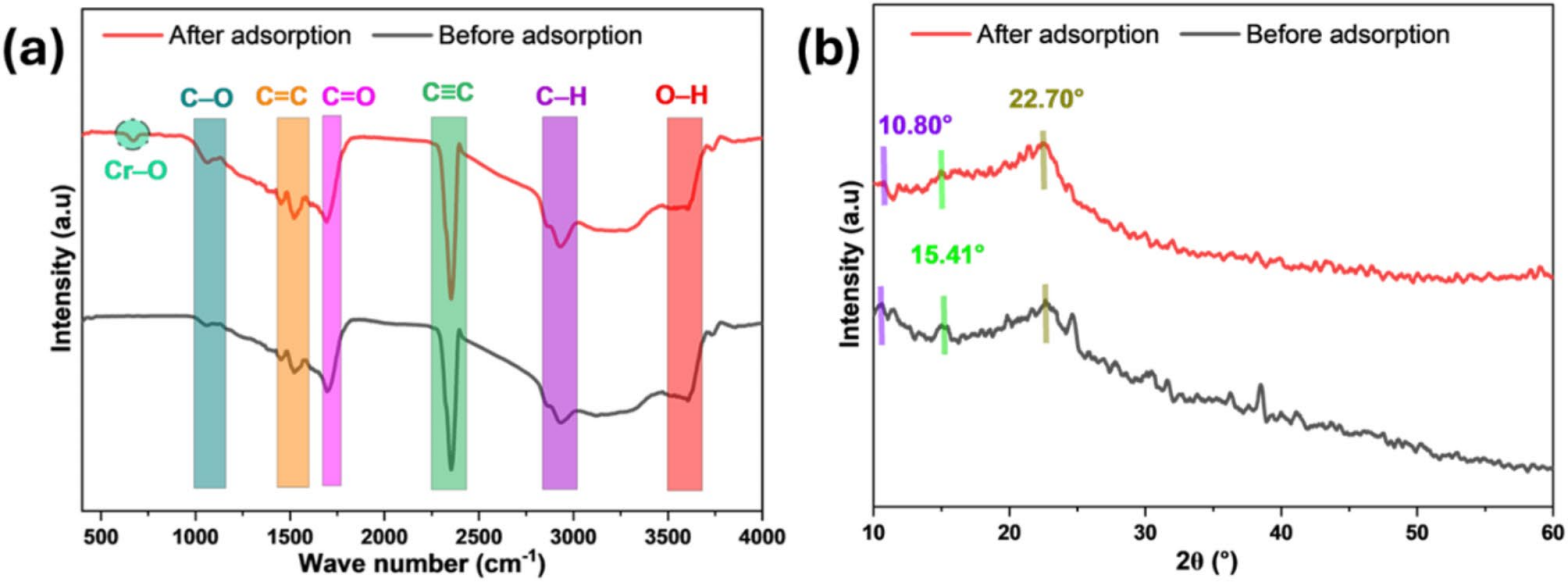
FTIR spectra (**a**) and XRD analysis (**b**) of AFHC adsorbent.

The FTIR analysis (Fig. 1a) of AFHC, prior to and after (VI) removal, revealed significant variations in the intensities of several peaks, implying the involvement of functional groups in the adsorption mechanism. The signal at 3605 cm^−1^, corresponding to O–H stretching vibrations^24^, exhibited a minor change after adsorption, suggesting the participation of hydroxyl groups through hydrogen bonding or electrostatic interactions with Cr(VI) ions. The 2972 cm^−1^ signal credited to aliphatic C–H stretching^25^, exhibited negligible change, implying minimal involvement of these groups. The C ≡ C bond appears as a sharp peak around 2353 cm^−1^ within the characteristic absorption region, indicating that the char contains a substantial amount of carbon^26^. The 1693.50 cm^−1^ signal, corresponding to C=O stretching vibrations^27^, decreased in intensity, suggesting that carbonyl groups participated in Cr(VI) binding, possibly through complexation or reduction. Similarly, the aromatic C=C stretching vibration at 1522 cm^−1^ exhibited reduced intensity^28^, indicating the partial involvement of aromatic rings, likely through π-electron interaction^29^. The 1051 cm^−1^ signal, associated with C–O stretching^28^, in alcohols, and ethers, showed a significant decrease, indicating active involvement of these groups in binding Cr(VI) through coordination or reduction mechanisms. Lastly, the 669 cm^−1^ signal, attributed to Cr–O vibrations, suggests to adsorption of chromium ions^30^.

XRD analysis serves as a crucial technique for examining the structural properties of AFHC used in adsorption studies. The XRD spectrum (Fig. 1b) of AFHC before Cr(VI) adsorption displays prominent peaks at 10.80°, 15.41°, and 22.70°, corresponding to the lattice planes (001), (101), and (002), respectively. The peak at 22.70° is characteristic of crystalline cellulose^31^. The peak at 10.80° is associated with the amorphous regions of cellulose and hemicellulose^32^. The peak at 15.41° is associated with hemicellulose, which exhibits a lower crystallinity degree than cellulose^33^. After adsorption, a shift in the peaks and changes in intensities were observed, suggesting expansion in the lattice structure as a result of Cr(VI) ion incorporation.

#### Surface characteristics and morphology assessment

**Fig. 2:**
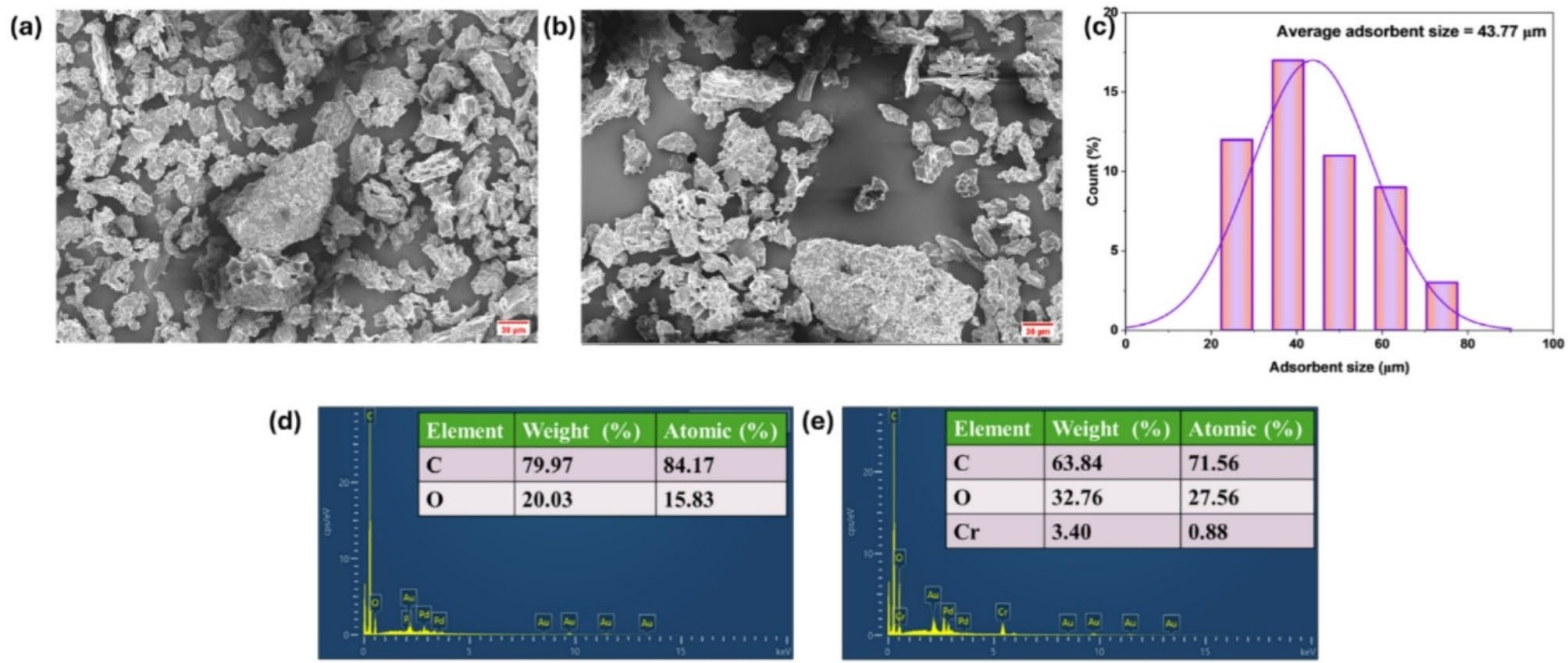
SEM micrographs before (**a**) and after Cr(VI) adsorption (**b**), distribution of AFHC size (**c**), EDS images before (**d**) and after Cr(VI) adsorption (**e**).

The SEM and EDS analyses provide valuable insights into the structural and compositional changes in AFHC before and after Cr(VI) removal. The SEM image of AFHC before adsorption (Fig. 2a) reveals an irregular structure with rough surfaces, suggesting a surface area conducive to adsorption^34^. The size distribution of AFHC is presented in Fig. 2c, illustrating the variation in particle sizes. The average particle size was found to be 43.77 μm, indicating a relatively uniform distribution. After Cr(VI) adsorption (Fig. 2b), noticeable morphological changes occur, with surface smoothening, indicating the successful attachment of Cr(VI) ions. EDS analysis further confirms the adsorption process, as the elemental composition shows a significant shift, with two distinct peaks for chromium observed at 0.56 and 5.4 keV following Cr(VI) uptake^35^. Initially, AFHC consists primarily of carbon (79.97 wt%) and oxygen (20.03 wt%) (Fig. 2d), reflecting a carbonaceous matrix with functional groups that facilitate adsorption. After Cr(VI) uptake (Fig. 2e), the oxygen content increases to 32.76 wt%, likely resulting from the reduction to Cr(III) and complex formation. The presence of Cr (3.40 wt%), post-adsorption provides direct evidence of chromium retention on the AFHC surface. These structural and compositional transformations suggest that Cr(VI) adsorption occurs through surface complexation and possible reduction mechanisms. Additionally, the observed agglomeration may result from chromium precipitation or strong electrostatic interactions between AFHC and Cr(VI) species^36^.

**Fig. 3:**
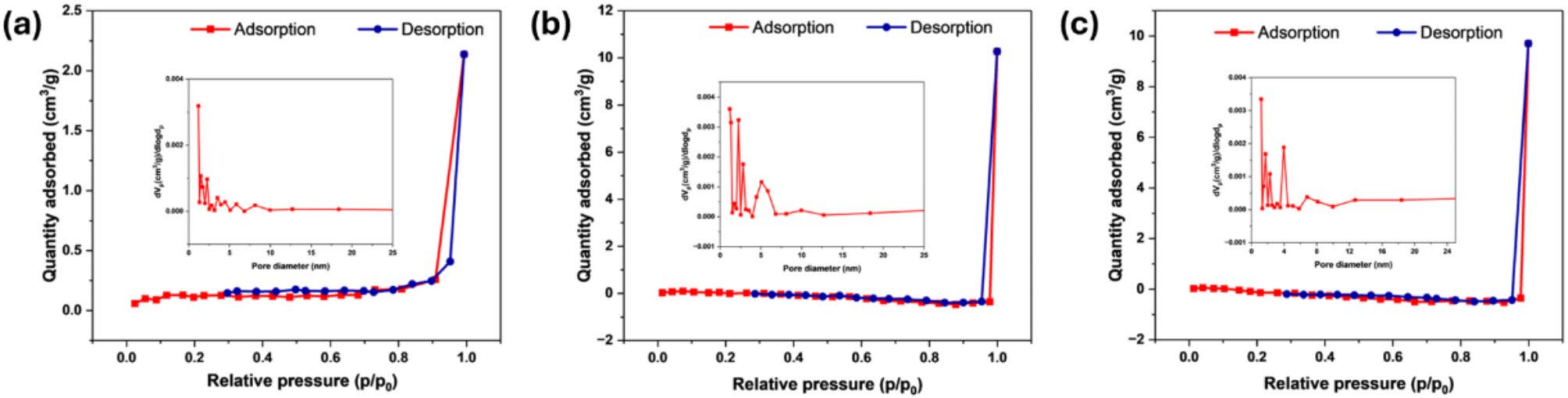
BET isotherm and pore size distribution of pristine biomass (**a**), AFHC before Cr(VI) adsorption (**b**), and AFHC after Cr(VI) adsorption (**c**).

The BET isotherm and pore size distribution of pristine *A. falcata* leaves biomass, AFHC and AFHC after Cr(VI) adsorption is presented in Fig. 3a, b and c, respectively. The N_2_ adsorption-desorption isotherm exhibited a Type IV, with a hysteresis loop, which is characteristic of mesoporous materials^37^. The *A. falcata* leaves biomass showed a relatively low SSA of 5.55 m^2^/g, a pore volume of 0.0032 cm^3^/g, and an average pore diameter of 1.51 nm. These values indicate a limited porous structure. Following hydrothermal carbonization, the biomass was converted into hydrochar (AFHC). The activation process resulted in a significant increase in SSA to 9.45 m^2^/g, with a corresponding increase in pore volume to 0.0087 cm^3^/g and a notable rise in pore diameter to 5.15 nm. The enhancement in these parameters suggests the successful formation of a more porous structure, which is beneficial for Cr(VI) adsorption. The increase in pore size and volume likely resulted from the removal of volatile components and the development of additional pore networks during the carbonization process^38^. After Cr(VI) adsorption, a decrease in SSA, pore volume, and pore diameter was observed. The SSA reduced to 8.46 m^2^/g, while the pore volume and pore diameter decreased to 0.0065 cm^3^/g and 3.99 nm, respectively. The reduction in these values can be attributed to the adsorption of Cr(VI) ions onto the surface and within the pores of AFHC, leading to partial pore blockage^39^.

#### XPS results

**Fig. 4:**
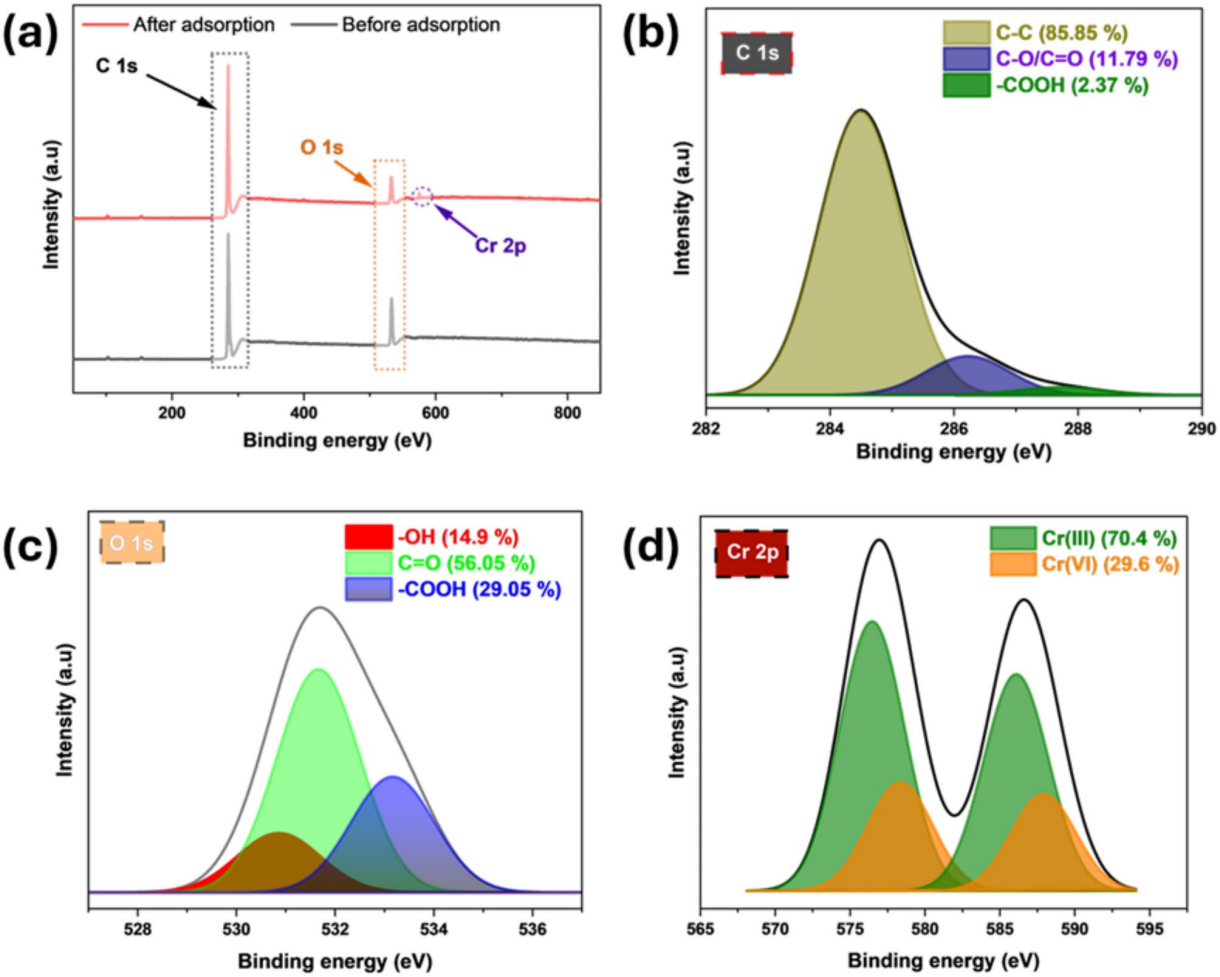
XPS survey spectrum (**a**) and deconvolution spectra of C1s (**b**), O1s (**c**), and Cr2p (**d**) for AFHC post-adsorption.

The C1s peak (Fig. 4a) was observed at 285.29 eV before adsorption. After adsorption, the C1s signal slightly shifted to a lower value of 285.05 eV. The slight shift indicates alterations in the surface chemistry of the AFHC, likely resulting from interactions of Cr(VI) species among carbon-based moieties, including hydroxyl, carboxyl, and aromatic structures. Similarly, the O1s peak before adsorption was recorded at 533.07 eV. Post-adsorption, the O1s signal moved to 532.5 eV. The shift indicates the involvement of oxygen-containing moieties during adsorption. The observed shifts in C1s and O1s binding energies confirm the binding of Cr(VI) onto AFHC. The reduction in oxygen peak intensity suggests the potential transformation of hexavalent to trivalent chromium. Two peaks corresponding to Cr 2p are observed at 577.04 eV (Cr 2p_3/2_) and 586.67 eV (Cr 2p_1/2_) after adsorption, further confirming the uptake and reduction of Cr(VI).

The post-adsorption high-resolution spectra (Fig. 4b-d) were analyzed, with deconvolution providing insights into functional group interactions and oxidation states. The C1s spectrum exhibited three peaks including a major peak at 284.49 eV (85.84%) that corresponds to C-C bonds, while the peaks at 286.23 eV (11.79%) and 287.72 eV (2.37%) represent respective C-O/C=O and -COOH groups^40^. For the O1s spectrum, three distinct peaks were identified that including a peak at 530.86 eV (14.90%) corresponds to -OH groups of aromatic and phenols, a peak at 531.67 eV (56.05%), assigned C=O groups, and the peak at 533.17 eV (29.05%), assigned to -COOH groups. The Cr2p spectrum confirmed the uptake and partial conversion of Cr(VI) to Cr(III). The signals at 578.35 eV (15.69%) and 587.93 eV (13.91%) represent Cr(VI), whereas the 576.48 eV (39.02%) and 586.10 eV (31.37%) signals correspond to Cr(III). The significant proportion of Cr(III) indicates the reduction of Cr(VI) on the AFHC surface, likely facilitated by oxygen-containing functional groups^41^. Both Cr(VI) and Cr(III) ions present in the solution form coordinated bonds with functional groups on the AFHC surface. This interaction leads to the formation of surface complexes, which are stable chemical species resulting from the binding of chromium ions to the reactive sites on AFHC.

### Adsorption analyses

#### Influence of solution pH

**Fig. 5:**
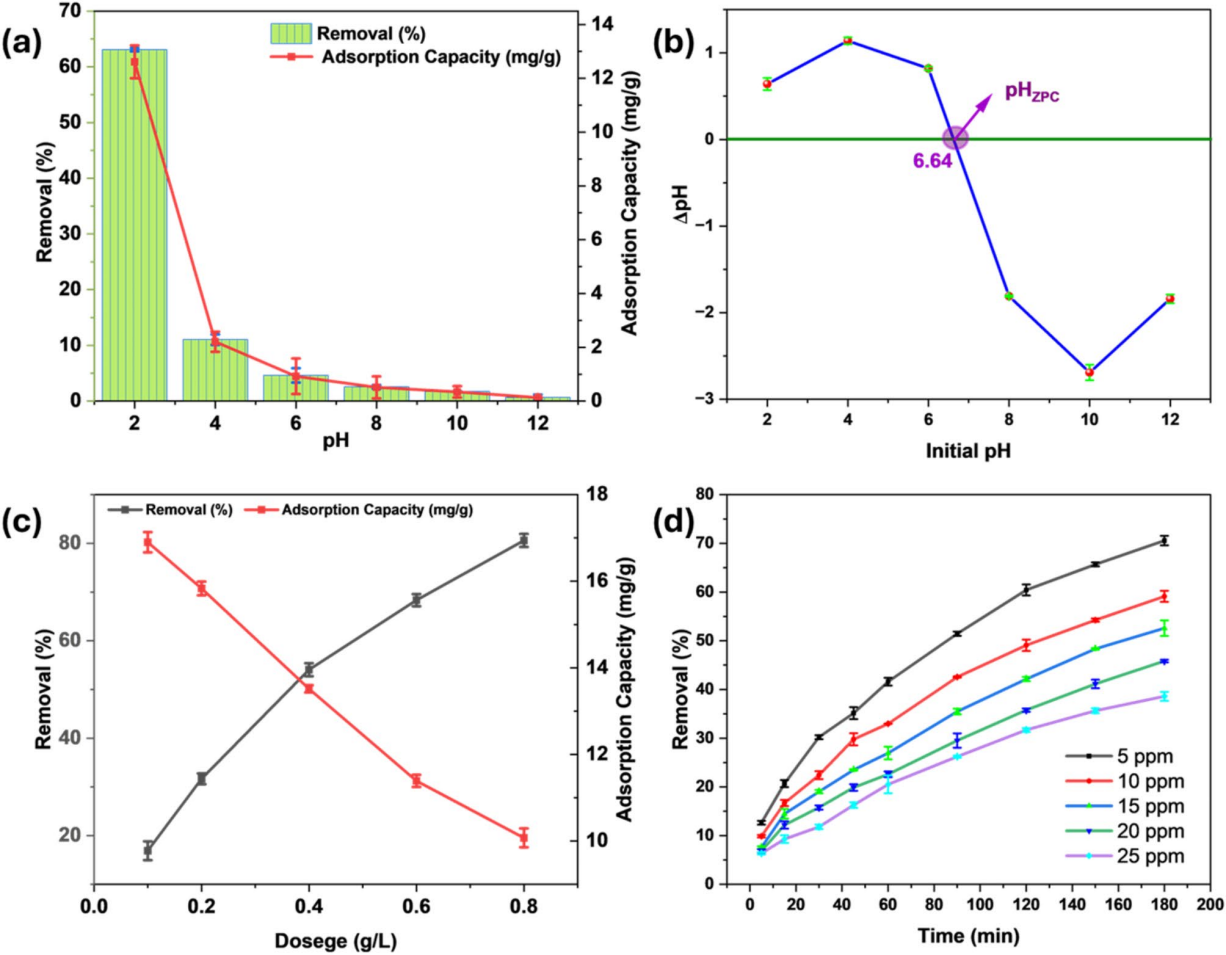
Effect of solution pH (**a**), zero point charge of AFAC (**b**), adsorbent dose (**c**), and Cr(VI) initial concentration with adsorption time (**d**).

Cr(VI) adsorption studies were conducted using a Cr(VI) concentration of 10 mg/L, an adsorbent dose of 0.5 g/L, and an orbital shaker set at 150 rpm, with all experiments performed at 303 K. The influence of pH on Cr(VI) adsorption and the adsorption capacity of AFHC was then assessed by varying the solution pH from 2 to 12 (Fig. 5a). At pH 2, the removal efficiency peaked at 63.07%, with an adsorption potential of 12.61 mg/g. At low pH values, particularly around pH 2, the dominant chromium species in solution are HCrO_4_^−^. The speciation of Cr(VI) is highly pH-dependent, influencing its interaction with the adsorbent surface. In acidic conditions, the equilibrium shifts towards the formation of HCrO_4_^−^ as the primary species due to the high concentration of H^+^ ions. In slightly less acidic environments, the equilibrium favors the coexistence of both HCrO_4_^−^ and Cr_2_O_7_^2−^. These negatively charged species (HCrO_4_^−^ and Cr_2_O_7_^2−^) are electrostatically attracted to the positively charged surface of AFHC at low pH, where the adsorbent surface is protonated, enhancing Cr(VI) removal efficiency^42^. At low pH, functional moieties on the AFHC surface, including carboxyl and hydroxyl groups, are protonated, creating a positively charged surface that enhances electrostatic attraction with Cr(VI) ions^43^. Furthermore, the redox potential of Cr(VI) is higher under an acidic environment, facilitating its reduction to Cr(III)^44^.

With the rise in pH from 2 to 12, both the removal performance declined sharply, with the efficiency dropping to 0.65% and the adsorption capacity reducing to 0.13 mg/g at pH 12. This reduction can be explained by three main factors. First, in alkaline conditions, the low concentration of H^+^ favors the deprotonation of HCrO_4_^−^ to CrO_4_^2−^, which predominates at high pH. However, the negatively charged AFHC surface due to functional group deprotonation leads to electrostatic repulsion with CrO_4_^2−^ ions and competition from OH^−^ ions for binding sites, resulting in reduced Cr(VI) removal^43^. Second, the redox potential of Cr(VI) decreases at high pH levels, reducing Cr(VI) to Cr(III) thermodynamically less favorable^45^. Third, under basic conditions, chromium species tend to form precipitates on AFHC, which hinders both the uptake and the reduction of Cr(VI)^46^. This diminished redox activity, combined with increased electrostatic repulsion and the precipitation of chromium oxides, leads to a significant drop in both sorption capacity and removal percentage. So, a solution pH of 2 was chosen for further experiments to optimize other parameters. The optimal pH of 2 observed in this study aligns with the results obtained using hydrochar derived from poultry litter^47^ and *Leersia hexandra* Swartz^48^, highlighting the importance of acidic conditions.

#### Zero-point charge

Below the zero-point charge (pH_ZPC_), the surface carries positively charged ions, enhancing anion adsorption, whereas above the pH_ZPC_, it becomes negatively charged, promoting cation adsorption^49^. The pH_ZPC_ of AFHC (Fig. 5b), determined through the pH drift method, was found to be 6.64, indicating that the AFHC has a neutral surface at this pH. The positive surface charge strengthens the electrostatic interactions of the AFHC with Cr(VI) moieties, including HCrO_4_^−^ and Cr_2_O_7_^2−^, which are predominant under acidic conditions. These interactions not only improve adsorption efficiency but also help the reduction^50^. On the other hand, at pH values above 6.64, the AFHC surface gains a negative charge from deprotonation, resulting in electrostatic repulsions with CrO_4_^2−^. Additionally, the reduced redox potential of Cr(VI) at elevated pH further decreases the adsorption efficiency^51^.

#### Dosage influence

The increase in AFHC dosage from 0.1 to 0.8 g/L, resulted in a proportional increase in Cr(VI) removal percentage, notably climbing from 16.89 to 80.58% (Fig. 5c). This improvement results from the greater availability of active sites at higher adsorbent concentrations, facilitating more Cr(VI) ion removal. As the adsorbent dose rises, more sites are exposed, providing greater opportunities for binding with Cr(VI) moieties. Consequently, this leads to higher Cr(VI) removal. The increased surface area and active sites make it easier for the adsorbent to capture more chromium ions. However, the sorption capacity reduced from 16.89 to 10.07 mg/g with an increasing dose. This drop is recognized to the lower Cr(VI) ion concentration in the solution, as fewer ions are present for adsorption. Additionally, the surplus of binding sites on AFHC leads to the underutilization of the AFHC’s capacity. As a result, the adsorption capacity of the adsorbent decreases. Therefore, 0.4 g/L was selected as the optimal dose.

#### Influence of Cr(VI) concentration and time

Batch studies were performed by maintaining the optimum AFHC concentration of 0.4 g/L constant. With the rise in the initial Cr(VI) concentration from 5 to 25 mg/L, the removal efficiency dropped from 70.55 to 38.59% (Fig. 5d). This decrease in removal efficiency is caused by the limited availability of active spots relative to the higher number of Cr(VI) ions in the solution^52^. At lower concentrations, the active spots are sufficient to capture the majority of Cr(VI) ions, resulting in higher removal performance. However, as the quantity of Cr(VI) ions rises, the adsorbent surface becomes saturated, leaving a greater fraction of Cr(VI) ions unadsorbed. Similar results have been reported for biochar-supported iron oxides^52^ and Cu/Fe bimetallic nanoparticles^53^.

The contact time influence using AFHC was investigated by varying the time from 5 to 180 min, as illustrated in Fig. 5d. The findings show that the removal efficiency improved as the contact duration increased for all concentrations. This trend is because of the increasing interaction of Cr(VI) ions with the active spots on the AFHC surface. During the initial phase, a swift rise in performance was observed due to the abundance of active spots and a steeper concentration gradient, which promoted faster adsorption^54^. As time progressed, the rate of Cr(VI) removal slowed down, as the active sites became increasingly occupied, the concentration gradient diminished, and the adsorbent neared saturation. This behavior aligns with observations for other adsorbents, including *Moringa oleifera* AC^54^ and date palm^55^.

#### Modelling of Cr(VI) adsorption

**Fig. 6:**
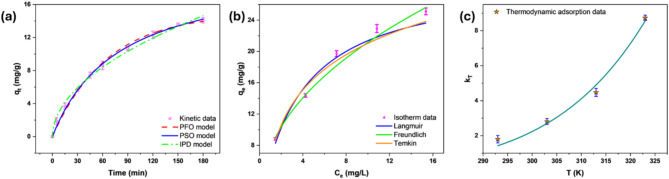
Cr(VI) adsorption onto AFHC: Kinetic models (**a**), isotherm models (**b**), and thermodynamic evaluation (**c**).

PFO, PSO, and IPD kinetic models were applied to analyze the binding of Cr(VI) onto AFHC at a concentration of 10 mg/L. The observed dynamic adsorption patterns and the associated model coefficients are depicted in Fig. 6a and detailed in Table 1, respectively. The correlation coefficient (R^2^) and chi-square (χ^2^) values were employed to assess the fit of each model.

**Table 1:** Model parameters for the kinetic, isotherm, and thermodynamic behavior of Cr(VI) removal in batch adsorption. (Kindly delete the empty cells/rows/columns. Align them properly. I could not edit the Table)

Kinetic models		
Kinetic model	Equation	Parameters
Pseudo First Order	${q}_{t}= {q}_{e }\left(1-exp\left({-k}_{1}t\right)\right)$	${k}_{1}$(min^− 1^) = 0.015
		${q}_{e}$(mg/g) = 14.80
		*R*^*2*^ = 0.993
		χ^2^ = 0.188
Pseudo Second Order	${q}_{t}=\frac{{q}_{e}^{2}{k}_{2}t}{{{q}_{e}K}_{2}t+1}$	${k}_{2}$(g/mg.min) = 0.007
		${q}_{e}$, (mg/g) = 19.95
		*R*^*2*^ = 0.997
		χ^2^ = 0.095
Intra-particle diffusion	${q}_{t}={k}_{p}{t}^{0.5}$+$C$	${k}_{p}$((mg/g) min^0.5^) = 1.118
		$C$(mg/g) = 0.279
		*R*^*2*^ = 0.994
		χ^2^ = 0.140

Isotherm models		
Isotherm	Equation	Parameters
Langmuir	${q}_{e}=\frac{{q}_{max}b{C}_{e}}{{(1+bC}_{e})}$	${q}_{max}$(mg/g) = 30.47
		*b* (L/mg) = 0.249
		*R*^*2*^ = 0.973
		χ^2^ = 19.50
Freundlich	${q}_{e}={K}_{F} {C}_{e}^{1/n}$	*K*_*F*_ ((mg/g)/(mg/L)^1/n^) = 7.486
		$n$ = 2.182
		*R*^*2*^ = 0.991
		χ^2^ = 7.012
Temkin	${q}_{e}=B ln(A{C}_{e})$	B = 6.684
		A (L/mg) = 2.429
		R^2^ = 0.979
		χ^2^ = 15.77

Thermodynamic model				
**T (K)**	293	303	313	323
**ΔG° (kJ/mol):**	-1.43	-2.60	-3.90	-5.82

Equation	Parameters
${k}_{T}=\text{exp}\left[\left(\frac{\Delta S^\circ }{R}\right)-\left(\frac{\Delta H^\circ }{R}\right)\frac{1}{T}\right]$	ΔH° (kJ/mol) = 46.75
	ΔS° (J/mol K) = 162
	*R*^*2*^ = 0.987
	χ^2^ = 0.179

q_e_: Equilibrium adsorption capacity; K_1_: PFO constant; K_2_: PSO constant; K: IPD rate constant; C: IPD intercept; q_max_: Monolayer adsorption capacity; b: Langmuir constant; K_F_: Freundlich constant; 1/n: adsorption intensity; C_e_: equilibrium Cr(VI) concentration; A & B: Temkin constants; ΔG°: (= – RT ln k_T_), standard Gibbs free energy; k_T_: (q_e_/C_e_), distribution factor; ΔH°: standard enthalpy and ΔS°: standard entropy.

The PSO model displayed the greatest R^2^ and smallest χ^2^ values (R^2^=0.997 and χ^2^ = 0.095), followed by the IPD model (R^2^ = 0.994 and χ^2^=0.140), and the PFO model (R^2^ = 0.993 and χ^2^ = 0.188). The PSO model’s fit indicates that the adsorption rate is proportional to the square of the number of unoccupied adsorption sites on the adsorbent surface, suggesting site occupation through bond formation or complexation over time. This implies that the adsorption process involves chemisorption, which includes the sharing or exchange of electrons between the Cr(VI) ions and the AFHC. This finding further supports the reduction of Cr(VI) to Cr(III) via surface moieties such as carboxyl and hydroxyl groups^56^. Literature extensively highlights the suitability of the PSO model to remove Cr(VI)^19,57^. Meanwhile, the PFO and IPD models highlight the roles of physisorption and diffusion, suggesting a multi-step Cr(VI) adsorption process involving surface adsorption, pore diffusion, and chemical interactions at the active sites of AFHC.

As illustrated in Fig. 6b, the findings indicate that the increase in the equilibrium Cr (VI) concentration improves the adsorption capacity of AFHC. This behavior occurs because, at lower Cr(VI) concentrations, there are abundant binding sites on the surface of AFHC relative to the number of Cr(VI) ions present. Consequently, the adsorption capacity is lower because the driving force for mass transfer between the solution and the adsorbent surface is relatively weak. This results in fewer interactions between Cr(VI) ions and the active sites on AFHC, leaving many sites unoccupied^58^. At higher equilibrium concentrations, the greater concentration gradient enhances mass transfer, increasing the collision frequency between Cr(VI) ions and the active sites on AFHC. This leads to more Cr(VI) species being adsorbed, progressively occupying the available binding sites. As a result, the adsorption capacity increases as the AFHC becomes more fully utilized, approaching its maximum adsorbent potential^59,20^.

The equilibrium dataset was analyzed with Langmuir, Freundlich, and Temkin isotherm models. Table 1 summarizes the isotherm parameters and corresponding statistical data. The Freundlich model demonstrated the best correlation with the experimental dataset, reflected by the highest R^2^ value (0.991) and the lowest χ^2^ value (7.012). This suggests that the adsorption is multilayered and occurs on a heterogeneous surface with varying affinities for Cr(VI). Additionally, the Freundlich exponent n was 2.182, which is greater than 1, signifying favorable adsorption.

The Langmuir model (R^2^=0.973, χ^2^=19.50) suggested monolayer adsorption as a possible mechanism, but its lower R^2^ and higher χ^2^ compared to the Freundlich model indicate deviations from its assumptions due to surface heterogeneity. Similarly, the Temkin model (R^2^=0.979, χ^2^=15.77) accounted for adsorbate-adsorbate interactions and exhibited a superior fit compared to the Langmuir model, but it remained less accurate than the Freundlich model. This further supports that the adsorption process is predominantly multilayered and takes place on a non-uniform surface. Furthermore, the adsorption capacity of AFHC, 30.47 mg/g remarkably exceeded the values reported in the literature by various adsorbents, as shown in Table 2.

**Table 2:** Adsorption effectiveness for Cr(VI) removal using diverse adsorbents.

Adsorbent	Synthesis method	S_a_ (m^2^/g)	pH	T(K)	Dose (g/L)	Time (min)	Cr (VI) (mg/L)	Adsorption capacity (mg/g)	Ref.
Paper sludge hydrochar	Hydrothermal carbonization	-	3	298	2	120	10–80	5.94	^67^
Rice husk magnetic biochar	Pyrolysis	134.6	2	351	0.5	120	1 − 10	9.97	^68^
*Acacia falcata* leaves magnetic nanoparticle	One pot synthesis	130.23	2	303	1.5	120	10 − 30	12.91	^69^
ZnCl_2_ modified hydrochar	Hydrothermal carbonization	26.5	5	298	0.1	360	5–15	14	^70^
Nitrogen-doped AC	Calcination	474.62	2	295	2	1440	20–40	15.15	^71^
Chitosan-based AC	Pyrolysis	1556	2	298	10	60	10–70	20.04	^72^
Melia azedarach wood magnetic biochar	Pyrolysis	5.22	3	298	5	540	5 − 200	25.27	^73^
*Acacia falcata* hydrochar	Hydrothermal carbonization	9.45	2	303	0.4	180	5–25	30.47	This study

By fitting the experimental data to the Van’t Hoff equation, the relationship between temperature and equilibrium constant is detailed in Fig. 6c, with model constants presented in Table 1. The high R^2^ value (0.987) and low χ^2^ value (0.179) confirm the thermodynamic model’s reliability. The equilibrium constant (k_T_), increased from 1.796 (293 K) to 8.74 (323 K), highlighting improved adsorption efficiency at higher temperatures. This rise suggests a favorable endothermic adsorption process. Higher temperatures increase the kinetic energy of Cr(VI) molecules, facilitating their movement. As a result, the diffusion rate of Cr(VI) onto the adsorbent surface improves. Consequently, adsorption capacity is enhanced at elevated temperatures^60^.

The Gibbs free energy (ΔG°) values, which become increasingly negative from − 1.43 kJ/mol at 293 K to − 5.82 kJ/mol at 323 K, confirm that the process occurs spontaneously and is thermodynamically advantageous at higher temperatures. The positive enthalpy change (ΔH°=46.75 kJ/mol) confirms the adsorption process is endothermic, enhancing Cr (VI) removal as temperature increases. The positive ΔH° value suggests that electrostatic interaction amongst Cr (VI) ion and the functional moieties on the AFHC surface primarily drive the adsorption. Furthermore, the findings align with the widely accepted thermodynamic threshold, as the ΔH° < 60 kJ/mol strongly indicates that the process is dominated by physical adsorption mechanisms, including electrostatic forces^61^. The positive change in entropy (ΔS°=162 J/mol K) implies greater randomness at the adsorbent-adsorbate interface resulting in the removal of water molecules and stronger Cr(VI)-AFHC interactions. Consistent thermodynamic patterns for Cr(VI) removal have been demonstrated across diverse adsorbents, such as pinecone biochar^21^, and polyethyleneimine functionalized magnetic char^62^.

### Regeneration studies

**Fig. 7:**
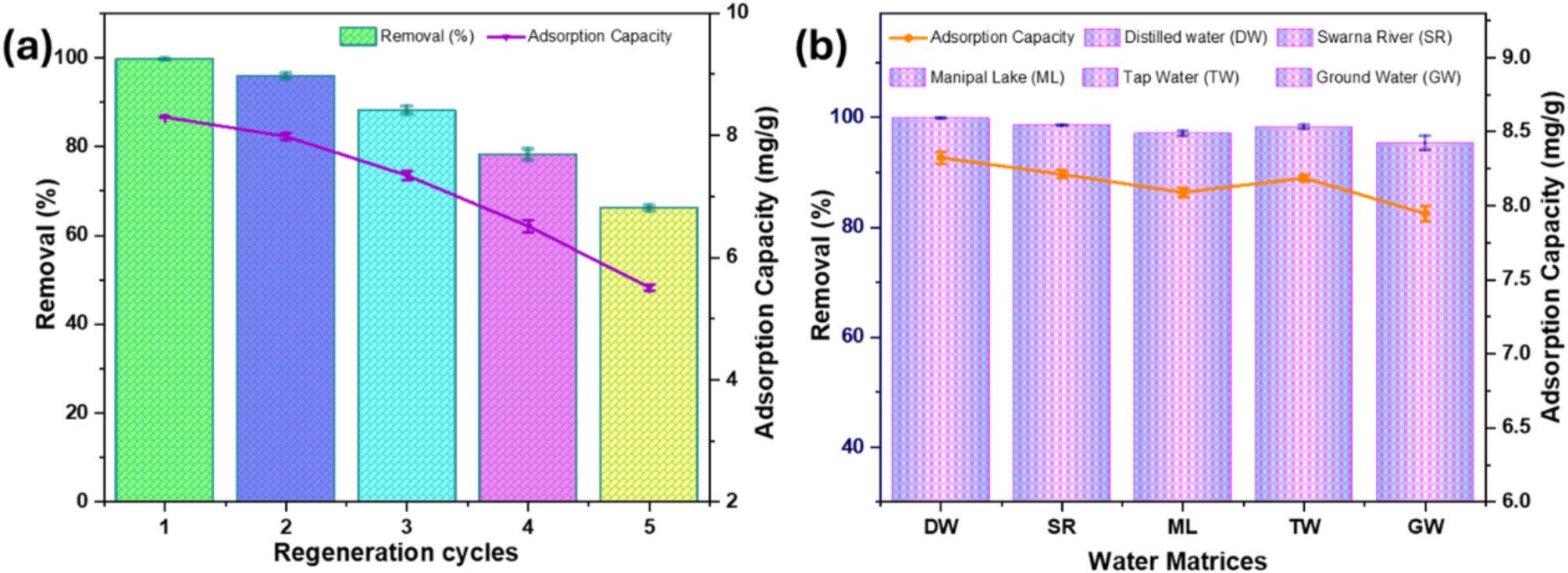
Study on AFHC regeneration (**a**) and its performance in treating Cr(VI)-spiked water sources (**b**).

Regeneration studies are essential for evaluating the reusability, stability, and long-term efficiency of an adsorbent in Cr(VI) removal. The regeneration potential of AFHC was assessed through five consecutive adsorption-desorption cycles, revealing a steady reduction in Cr(VI) removal performance (Fig. 7a). Initially, the adsorption removal was 99.59%. Nonetheless, after five cycles, the removal efficiency fell to 66.12%. This reduction can be attributed to pore blockage, and loss of active functional groups, during the regeneration process^63^. Despite this decline, AFHC retained a removal efficiency of over 78.17% after four cycles, demonstrating its potential for reuse up to four times. These results emphasize the significance of regeneration studies in evaluating the practical feasibility of AFHC for large-scale Cr(VI) removal applications, emphasizing the need for further improvements to enhance its reusability and long-term performance.

### Investigation of AFHC’s performance in removing Cr(VI) from spiked water matrices

Spiked studies are critical in assessing the efficacy of adsorbents in practical water treatment applications, as they simulate the complex and dynamic conditions of natural water sources. By spiking various water matrices such as distilled water, Swarna River, tap water, Manipal Lake, and groundwater with Cr(VI), these studies allow for assessment of how interfering ions, organic and inorganic molecules, and other contaminants impact the effectiveness. Herein, (Fig. 7b), the removal percentage of AFHC was higher in distilled water (99.89%) compared to other water sources, where the removal percentage ranged from 95.35 to 98.55%. The decrease in removal performance is linked to the existence of competing ions, and organic and inorganic materials in the diverse water sources. These findings highlight the significance of spiked studies in assessing the practical feasibility of adsorbents, providing a more accurate representation of their behavior in diverse water sources.

### Cr(VI) removal mechanism

**Fig. 8:**
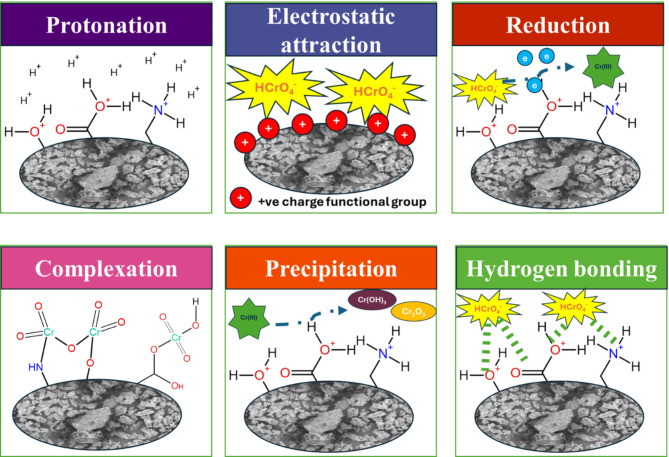
Mechanistic insights into Cr(VI) removal by AFHC.

The performance of AFHC for Cr(VI) removal is significantly influenced by its physical and chemical properties, as well as the underlying adsorption mechanism, as depicted in Fig. 8. The irregular surface morphology of AFHC, observed in SEM images, provides abundant adsorption sites, while EDS confirms the presence of chromium at 0.57 keV and 5.41 keV after adsorption, indicating successful chromium uptake^35^. At pH 2, the protonation of surface functional moieties, including hydroxyl and carboxyl groups, enhances electrostatic attraction between the positively charged AFHC surface and negatively charged Cr(VI) species, facilitating the initial binding of Cr(VI) onto AFHC^43^. This aligns with previous studies showing that electrostatic interactions play a crucial role in the adsorption of anionic species under acidic conditions.

The chemical transformations of Cr(VI) on AFHC are supported by FTIR, XRD, and XPS analyses. FTIR spectra showed shifts in characteristic peaks corresponding to hydroxyl, and carboxyl, groups, indicating their involvement in Cr(VI) reduction and complexation^64^. XPS analysis provided direct indications of Cr(VI) reduction, as shown by the deconvoluted Cr 2p spectrum. The presence of peaks at 578.35 eV (15.69%) and 587.93 eV (13.91%) confirmed the binding of Cr(VI) species, while the peaks at 576.48 eV (39.02%) and 586.10 eV (31.37%) corresponded to Cr(III), indicating partial reduction^41^. The dominance of Cr(III) suggested that a significant amount of Cr(VI) was reduced upon interaction with AFHC surface functional groups^65^. This reduction process is crucial as Cr(III) forms stable complexes and precipitates as Cr(OH)_3_, minimizing its mobility and toxicity in aqueous systems.

The presence of newly formed Cr-O bonds in the FTIR spectra at 669 cm^−1^ confirms inner-sphere complexation between chromium species and AFHC’s oxygen-containing functional moieties^30^. Additionally, FTIR spectra exhibit broadening and shifts in O-H stretching vibrations, indicating the involvement of hydrogen bonding between chromium species and surface functional groups^66^. These interactions, coupled with the structural and chemical modifications observed through analytical techniques, confirm a multi-step adsorption mechanism, including protonation and electrostatic attraction, Cr(VI) reduction, chromium precipitation, complexation, and hydrogen bonding. This comprehensive mechanism highlights AFHC’s efficiency as a potential adsorbent to remediate Cr(VI) contamination.

## Conclusions

This study demonstrated the successful synthesis of AFHC from *A. falcata* leaves via hydrothermal carbonization for efficient Cr(VI) remediation. Adsorption efficiency was significantly influenced by pH, with maximum removal observed at pH 2 due to enhanced electrostatic attraction between Cr(VI) ions and surface functional groups. Kinetic data followed the pseudo-second-order model, indicating chemisorption, while the Freundlich isotherm confirmed heterogeneous adsorption. The highest adsorption capacity was 30.47 mg/g at 303 K, and thermodynamic analysis indicated a spontaneous, endothermic process. SEM revealed an irregular, rough surface structure, and EDS showed high oxygen content (32.76 wt%), linked to functional groups involved in adsorption. FTIR and XPS analyses confirmed the role of oxygen-rich functional groups in Cr(VI) reduction and complexation, with partial transformation to Cr(III). Regeneration studies confirmed usability for up to four cycles, and spiked studies validated its effectiveness in real-world water samples. These findings establish AFHC as a novel, sustainable adsorbent for Cr(VI) removal from wastewater.

## Data Availability

The authors declare that the data supporting the findings of this study are available within the article.
